# mtDNA nt13708A Variant Increases the Risk of Multiple Sclerosis

**DOI:** 10.1371/journal.pone.0001530

**Published:** 2008-02-13

**Authors:** Xinhua Yu, Dirk Koczan, Anna-Maija Sulonen, Denis A. Akkad, Antje Kroner, Manuel Comabella, Gianna Costa, Daniela Corongiu, Robert Goertsches, Montserrat Camina-Tato, Hans-Juergen Thiesen, Harald I. Nyland, Sverre J. Mørk, Xavier Montalban, Peter Rieckmann, Maria G. Marrosu, Kjell-Morten Myhr, Joerg T. Epplen, Janna Saarela, Saleh M. Ibrahim

**Affiliations:** 1 Section of Immunogenetics, University of Rostock, Rostock, Germany; 2 Department of Immunology, University of Rostock, Rostock, Germany; 3 Department of Molecular Medicine, National Public Health Institute, Helsinki, Finland; 4 Department of Human Genetics, Ruhr University, International Graduate School of Neuroscience (IGSN), Bochum, Germany; 5 Clinical Research Group for Multiple Sclerosis and Neuroimmunology, Department of Neurology, University of Würzburg, Wüzburg, Germany; 6 Unitat de Neuroimmunologia Clinica, Hospital Universitari Vall d'Hebron, Universitat Autonoma de Barcelona, Barcelona, Spain; 7 The Centro Sclerosi Multipla, Dipartimento di Scienze Cardiovascolari e Neurologiche, University of Cagliari, Cagliari, Italy; 8 Department of Neurology, Haukeland University Hospital, University of Bergen, Bergen, Norway; 9 Department of Clinical Medicine, University of Bergen, Bergen, Norway; 10 Department of Pathology, Haukeland University Hospital, Gade's Institute, University of Bergen, Bergen, Norway; Stanford University, United States of America

## Abstract

**Background:**

Mitochondrial DNA (mtDNA) polymorphism is a possible factor contributing to the maternal parent-of-origin effect in multiple sclerosis (MS) susceptibility.

**Methods and Findings:**

In order to investigate the role of mtDNA variations in MS, we investigated six European MS case-control cohorts comprising >5,000 individuals. Three well matched cohorts were genotyped with seven common, potentially functional mtDNA single nucleotide polymorphisms (SNPs). A SNP, nt13708 G/A, was significantly associated with MS susceptibility in all three cohorts. The nt13708A allele was associated with an increased risk of MS (OR = 1.71, 95% CI 1.28–2.26, *P* = 0.0002). Subsequent sequencing of the mtDNA of 50 individuals revealed that the nt13708 itself, rather than SNPs linked to it, was responsible for the association. However, the association of nt13708 G/A with MS was not significant in MS cohorts which were not well case-control matched, indicating that the significance of association was affected by the population structure of controls.

**Conclusions:**

Taken together, our finding identified the nt13708A variant as a susceptibility allele to MS, which could contribute to defining the role of the mitochondrial genome in MS pathogenesis.

## Introduction

Multiple sclerosis (MS) is a chronic inflammatory neurodegenerative disease that affects the central nervous system in genetically susceptible individuals. The mitochondrial genome (mtDNA) has been suggested to be involved in MS development for several reasons. First, mtDNA variations play an important role in neurodegenerative diseases, with deleterious mtDNA mutations causing early onset neurodegenerative disorders, and common mtDNA polymorphisms are associated with late-onset neurodegenerative disorders [1]–[5]. Secondly, Lebeŕs hereditary optic neuropathy (LHON), a disease caused by mtDNA mutations, includes symptoms of inflammatory demyelination similar to MS [6]. Those observations suggest that mtDNA could play a role in pathogenesis of MS. This hypothesis is also supported by the observations from two family-based half-sibling studies, suggesting that there is a maternal parental-of-origin effect in MS [7], [8]. The search for the mtDNA variations contributing to MS started about a decade ago [9], [10], and most studies focused on the LHON mutations. The consensus is that the LHON primary mutations are not associated with MS [11]. LHON secondary mutations, such as nt4216 and nt13708, and their related haplogroup J, however, were suggested to be associated with MS [10], [12]–[14]. But because of the small number of samples used, these associations are rather weak or even debatable [9], [11], [14].

A common difficulty in investigating the role of mtDNA in polygenetic diseases is that it is challenging to precisely define the susceptibility SNP. Since human mtDNA is characterized by maternal transmission and a lack of recombination, it always exist in haplotype forms [15]. Therefore, associations between mtDNA polymorphisms and polygenic diseases are always identified as associations between mtDNA haplotypes and diseases, making it challenging to precisely define the mtDNA polymorphism contributing to diseases[5], [16]. There could be two solutions to this. One is to evaluate the association between each mtDNA polymorphism on the haplotype and the disease. This method is powerful but extremely resource-consuming. The second method is much less resource consuming but is only suitable for some cases. That is, when two related mtDNA haplotypes were associated with the same diseases, to check the mtDNA polymorphism(s) shared by these two haplotypes. Nevertheless, for both methods, a large number of samples are required.

In this study, we collected six European MS cohorts comprising >2,500 sporadic cases and a similar number of healthy controls. The aim of this study is twofold. The first one is to investigate whether the mtDNA polymorphisms or haplotypes are associated with the susceptibility to MS. Secondly, if there is an association, we attempt to identify the susceptibility mtDNA polymorphism using this larger number samples.

## Results

### nt13708 G/A is associated with susceptibility to MS

Previously, a maternal parent-of origin effect was observed in a large half-sibling study in MS family [8]. This suggests a role of mtDNA variations in MS, and the susceptibility variation could be a common mtDNA polymorphism. Based on this, we selected seven potentially functional common mtDNA polymorphisms with frequencies of more than 3%. In addition, the three Caucasian mtDNA haplogroups, J, K, and I, that are related to polygenic diseases, could be constructed with these seven SNPs [16]. We collected six European MS cohorts containing ∼5,000 samples. In the initial part of the study, we genotyped seven mtDNA polymorphisms in three well matched cohorts, in terms of both age and geography. The frequencies of nt13708 minor allele, A, were significantly higher in MS cases than in controls in all three cohorts, with OR values of 1.63 to 1.82. Meta-analysis of the combined cohorts showed that nt13708A variant was significantly associated with an increased risk to MS (OR = 1.71, 95% CI 1.29–2.27, P = 0.0002). The six other SNPs were not associated with MS (Table 1). Three European mtDNA haplogroups could be constructed with the seven SNPs used in this study according to Torroni et al [17], [18], including haplogroups I, J and K. The frequency of haplogroup J, a haplogroup constructed with nt10398 and nt13708 SNPs, was higher in MS cases than in controls (OR = 1.53, 95% CI 1.13–2.09, *P* = 0.007), with the same tendency in all three cohorts. Haplotype I and K were not associated with MS (Table 1).

**Table 1:** Associations between mtDNA polymorphisms and susceptibility to MS in three European cohorts

SNP	Gene	Spain					Norway					Germany I					Pooled*		
		Frequency		OR	95% CI	P	Frequency		OR	95% CI	P	Frequency		OR	95% CI	P	OR	95% CI	P
		Patient (n = 417)	Control (n = 510)				Patient (n = 390)	Control (n = 190)				Patient (n = 285)	Control (n = 382)						
1719 G/A	16S rRNA	0.036	0.029	1.23	0.55–2.73	0.581	0.077	0.074	1.05	0.52–2.19	1	0.035	0.039	0.89	0.35–2.15	0.839	1.06	0.70–1.01	0.875
4216 T/C	ND1 H/Y	0.177	0.137	1.36	0.93–1.97	0.101	0.215	0.158	1.46	0.91–2.40	0.119	0.228	0.230	0.98	0.67–1.44	1	1.22	0.99–1.53	0.078
9055 G/A	ATP6 A/T	0.082	0.075	1.10	0.66–1.84	0.712	0.062	0.079	0.77	0.37–1.61	0.481	0.060	0.055	1.09	0.53–2.22	0.866	1.00	0.72–1.41	0.952
10398 A/G	ND3 T/A	0.211	0.178	1.23	0.88–1.73	0.241	0.200	0.205	0.97	0.62–1.53	0.912	0.147	0.139	1.07	0.67–1.70	0.822	1.11	0.89–1.39	0.377
**13708 G/A**	**ND5 A/T**	**0.124**	**0.080**	**1.63**	**1.04–2.58**	**0.028**	**0.185**	**0.111**	**1.82**	**1.06–3.23**	**0.022**	**0.116**	**0.071**	**1.72**	**1.01–2.93**	**0.047**	**1.71**	**1.29–2.27**	**0.0002**
16189 T/C	D-loop	0.119	0.139	0.84	0.56–1.26	0.433	0.105	0.079	1.37	0.72–2.74	0.370	0.133	.0115	1.18	0.72–1.93	0.477	1.03	0.79–1.35	0.852
16223 C/T	D-loop	0.160	0.171	0.89	0.62–1.29	0.592	0.153	0.184	0.81	0.50–1.32	0.403	0.123	0.141	0.85	0.52–1.37	0.565	0.86	0.68–1.09	0.236
Haplogroup																			
I		0.017	0.016	1.07	0.33–3.41	1	0.023	0.037	0.62	0.20–1.99	0.418	0.014	0.021	0.66	0.15–2.51	0.570	0.77	0.42–1.43	0.499
**J**		**0.110**	**0.071**	**1.63**	**1.01–2.66**	**0.037**	**0.125**	**0.084**	**1.56**	**0.84–3.03**	**0.161**	**0.087**	**0.065**	**1.37**	**0.73–2.55**	**0.300**	**1.54**	**1.13–2.09**	**0.007**
K		0.062	0.063	0.99	0.56–1.75	1	0.043	0.042	1.04	0.41–2.83	1	0.021	0.037	0.57	0.18–1.59	0.262	0.90	0.60–1.35	0.688

* meta-analysis for pooled sample were performed using Mantel-Haenszel test. Halpogroups I, K and J were constructed with 10398G-1719A, 10398G-9055A and 10398G-13708A respectively.

### nt13708 G/A is the variation responsible for the association

**Figure 1:**
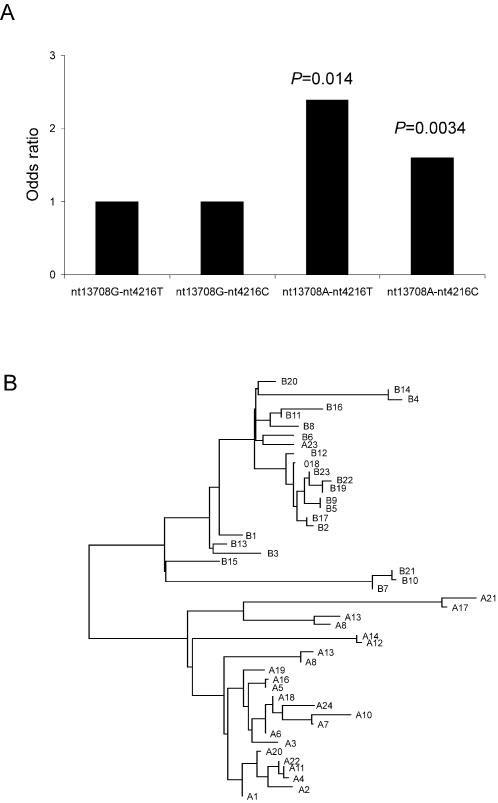
Two mtDNA haplotypes associated with MS. (A) Evaluation of the association between four mtDNA haplotypes and MS. The four haplotypes were constructed with nt13708 G/A and nt4216 T/C polymorphisms. The association was evaluated using the wildtype haplotype (nt13708G-nt4216T) as the control genotype, and Odds ratios and *P* values of the other three haplotypes were determined. (B) Phylogenetic tree of the two disease-associated mtDNA hyplotypes. The phylogenetic tree were constructed base on the mtDNA sequence of 24 sample with nt13708A-nt4216C (sample A1–A24) and 23 samples with nt13708A-nt4216T (sample B1–B23) using ClusterW softeware.

The nt13708A allele was reported to be linked to nt10398G and nt4216C alleles and regarded as a tag-SNP of the haplogroup J [17], [18]. In this study, we observed that most of the nt13708A alleles were linked to the nt10398A and nt4216C alleles (∼85%). However, a small proportion of nt13708A alleles (∼15%) were not. Thus, the mtDNA carrying nt13708A allele could be divided into two haplotypes, nt13708A-nt4216C (haplogroup J) and nt13708A-nt4216T. We then determined whether the both haplotypes are associated with MS. Both haplotypes, nt13708A-nt4216C (OR = 1.60, 95% CI 1.18–2.17, *P* = 0.00349) and nt13708A-nt4216T (OR = 2.39, 95% CI 1.22–4.72, *P* = 0.014) significantly increased the risk of MS as compared to the wildtype haplotype, nt13708G-nt4216T. (Figure 1A).

The two disease associated mtDNA haplotypes make it possible to identify the SNP responsible for the association by checking the variation(s) shared by then. We subsequently sequenced the complete mitochondrial genome of 50 individuals, containing 24 samples with nt13708A-nt4216C, 23 samples with nt13708A-nt4216T and three samples with nt13708G. Samples were randomly selected from patients and controls. The phylogenetic analysis showed that samples with nt13708A-nt4216C and nt13708A-nt4216T haplotypes formed two distinct branch on the phylogenetic tree, indicating that they are differed from each other considerably (Figure 1B). We then checked overlap variants shared by both haplotypes. Although a few polymorphisms, e.g. nt2706 A/G and nt7028 C/T, carrying by one haplotype also existed in several cases of the other haplotype, the only SNP shared by the two susceptibility haplotypes was nt13708 G/A (Table 2). Therefore, nt13708 G/A, rather than an SNP linked to it, was the polymorphism contributing to the susceptibility to MS.

**Table 2:** mtDNA variations linked to in nt13708A variant

Variations*	Gene	Frequency in haplotype nt13708A-nt4216C	Frequency in haplotype nt13708A-nt4216T
73 a/g	Control region	14/24	4/23
185 g/a	Control region	7/24	0/23
195 t/c	Control region	6/24	5/23
228 g/a	Control region	12/24	0/23
295 c/t	Control region	23/24	0/23
489 t/c	Control region	23/24	0/23
1438 g/a	12S rRNA	0/24	9/23
2706 a/g	16S rRNA	23/24	7/23
3010 g/a	16S rRNA	19/24	3/23
4216 t/c	Mt-ND1 Y-H	24/24	0/23
4769 g/a	Mt-ND2 syn	0/24	9/23
7028 c/t	Mt-COI syn	23/24	7/23
10398 a/g	Mt-ND3 T-A	23/24	0/23
11251a/g	Mt-ND4 syn	23/24	0/23
11719 g/a	Mt-ND4 syn	23/24	4/23
12612 a/g	Mt-ND5 syn	23/24	0/23
**13708 g/a**	**Mt-ND5 A-T**	**24/24**	**23/23**
14766 c/t	Mt-CYB T-I	23/24	4/23
14798 t/c	Mt-CYB F-L	17/24	0/23
15452 c/t	Mt-CYB L-I	23/24	0/23
16069 c/t	Control region	23/24	0/23
16126 t/c	Control region	23/24	0/23
16519 t/c	Control region	1/24	9/23

* Positions of variations are according to the reference sequence J01415. Only those variations with frequencies of more than 20% in the total 50 sequenced samples are presented.

### The association is not significant in non-well matched cohorts

We then investigated the association of nt13708A variant with MS in other three European cohorts which were not case-control well matched, in terms of the age or geography (Table 3). However, no significant association was observed (Table 4). Previous studies showed that the frequency of nt13708A variant (haplogroup J) was associated with age, with higher frequency in centenarians than in younger individuals[19], [20]. Also, its frequency varied considerably among populations [5], [9], [14], [21]–[24]. We proposed that the lack of significant association was due to the difference between cases and controls in age or geographic origin.

**Table 3:** Population demographics of the non-well matched MS cohorts

Cohorts	Patients			Controls			
	Number	Age (mean±SD)	Location	Number	Age (mean±SD)	Location	Composition
Finland ξ	936	42.9±10.0	Finland	970	63.1± 12.4 ζ *	Finland	Unrelated controls
							Father of controls
							Father of patients
Germany II	342	38.1±10.5	Bochum	371	43.7±12.4	Essen	Unrelated controls
Sardinia	194	37.8±10.2	Sardinia	194	62.5±15.5*	Sardinia	Father of the patient

ξ The samples of the Finnish cohort have been collected from five university and central hospitals around Finland (Helsinki, Tampere, Seinäjoki, Kuopio, Oulu).

ζ , calculated from 700 samples with age infomation.

* , significantly higher than patients.

**Table 4:** Non-significant association of nt13708A variant with MS in three cohorts

	Frequency		OR (95% CI)
	Patient	Control	
Finland* §	0.068 (n = 936)	0.059 (n = 980)	1.18 (0.75–1.95)
Germany II§	0.137 (n = 342)	0.132 (n = 371)	1.04 (0.67–1.61)
Sardinian*	0.165 (n = 194)	0.134 (n = 194)	1.27 (0.72–2.23)

* cases and controls are not age matched and

§ cases and controls are not geographic matched.

To test this, we performed further analysis with the large Finnish cohort by stratifying the samples. All samples were classified into two groups according to the geographic origin. Finland I group contained samples from the Southern Ostrobothnia region where the incidence of MS is higher than other Finnish regions [25], and Finland II group contained samples from other regions. In each geographic group, controls were further stratified into two group according to age. Indeed, when we classified the cohort, we observed that the frequency of nt13708A variant was affected by, both, age and geographic origin. The frequency of the nt13708A variant was higher in the Finland I cohort than in the Finland II cohort (7.1% *vs.* 5.1% in control and 10.2% *vs.* 5.9% in patients). Also, the frequency of nt13708A was associated with age, with lower frequency in unrelated controls than their fathers in Finland II cohort (3.3% *vs.* 5.5%). When using the younger, unrelated, geographically matched controls, the odds ratio increased to 1.84 which was similar to those three well matched MS cohorts (Table 5). Although the association was still not significant because of the small number of samples after stratification, this suggests that association of nt13708 G/A with MS could be affected by the population structure of the cohort.

**Table 5:** Association of nt13708A variant with MS in Finnish sub-cohorts

	Finland I §			Finland II ξ		
	Frequency		OR (95% CI)	Frequency		OR (95% CI)
	Patient	Control		Patient	Control	
Non-age matched, controls younger*	-	-	-	0.059 (n = 730)	0.033 (n = 122)	1.84 (0.65–5.19)
Non-age matched, controls older **	0.102 (n = 206)	0.071 (n = 435)	1.48 (0.83–2.64)	0.059 (n = 730)	0.056 (n = 413)	1.06 (0.63–1.77)

§ , Southern Ostrobothnia region;

ξ , Finland except Southern Ostrobothnia region.

* , the controls in Finland II cohort consist of 122 unrelated controls.

** For the Finland I cohort, the controls consists of 70 fathers of the patients (69.1± 11.0 years) and 365 unrelated controls (59.7±18.7 years) which are significantly higher than patients (46.3±11.7 years). For the Finland II cohort, controls consist of 295 fathers of the patients (66.8±9.5 years) and 108 fathers of the unrelated controls.

## Discussion

In this study, we identified an association between nt13708 G/A polymorphism and susceptibility to MS, which was consistent with previous results [10], [12]. By sequencing mtDNA of samples with two disease association haplotypes, we demonstrated that nt13708A variant was responsible for the increase of the risk of MS. To our knowledge, this is the first time to identify a casual variation on mtDNA predisposing polygenic disease in human. This precise identification will help in exploring the molecular mechanisms of pathogenesis of MS. Also, since the nt13708 G/A polymorphism is a tag-SNP of haplogroup J which has been reported to be associated with other complex clinical traits, *e.g.* increasing the risk of type 2 diabetes and longevity [20], [24], it is reasonable to speculate that nt13708A variant might be responsible for the effect.

The nt13708 G/A SNP is located in the *mt*-*ND5* gene. It results in an amino acid substitution of A to T at a moderately conserved region. A recent study investigating the functions of the nt4216 T/C and nt13708 G/A mutations revealed that they do not further impair *in vivo* mitochondrial oxidative metabolism when linked woth the primary LHON mutation nt11778 [26]. However, the function of the nt13708 G/A itself has not been investigated. Given the role of nt13708A in the MS, investigating the function of this common variant which will help to elucidate the pathogenesis of the disease. In the future, we plan to approach this aim using two strategies. First, study the mitochondrial performance in large numbers of patients and controls to compensate for the genetic heterogeneity. The second strategy is using mouse model. Since most mouse classic inbred strains (CIS) were descended from a single female and mtDNA mutation are common in CIS, it is possible to identify strains whose mtDNAs differ from each other only in a mt-ND5 mutation [27]. Thereafter, conplastic strains for such mutation could be generated and used to investigate the role of the mt-ND5 in mouse models of MS.

Although the mechanism underlying the effect of nt13708A variant on MS is still unclear, several recent publications demonstrating the association of MS or its animal model with mitochondrial genes e.g. uncoupling protein 2 (UCP2) suggest a potential mechanism. UCP2 is located in the mitochondrial inner membrane and uncouples the protons generated during oxidative phosphorylation (OXPHOS) [28]. UCP2 has been reported to play a role in MS and its animal model, Experimental Autoimmune Encephalomyelitis (EAE), and its role in affecting reactive oxygen species (ROS) production in mitochondria has been proposed to be a potential mechanism [28]–[30]. Therefore, it is conceivable that mt-ND5 nt13708A variant might increase the susceptibility to MS by affecting the mitochondrial ROS production.

It is noteworthy to mention the heterogeneity in the significance of the association of nt13708 G/A polymorphism with MS. Such heterogeneity was also observed among previous studies with relative small number of the samples [9], [10] [12]. However, due to the small sample size, this heterogeneity could not be evaluated genetically. In this study, with a large sample size in Finnish cohort, we showed that age and geographic origin of controls could be the reason for the heterogeneity. The frequency of nt13708A variant or J haplogroup have be reported to be associated with aging in Finnish and Sardinian population observations, with higher frequency in the older than the younger people [19], [20]. This was confirmed in our study in both Finland II cohort (5.5% vs 3.3%) and Sardinia cohort (13.4% vs 7.8% [20], [23]). Therefore, when the controls are considerably older then the cases, the significance of the association between nt13708 G/A and MS will be reduced. Also, we observed that the frequency of the nt13708A variant is associated with geographic origin in the Finnish cohort. The frequency of the nt13708A variant was higher in the Southern Ostrobothnia region where the prevalence of MS disease is comparatively high [23], [25] than in the rest of Finland. Thus, when samples are collected from different geographic origins that differ from each other in the frequency of nt13708A variant, the significance of the association will be affected.

**Figure 2:**
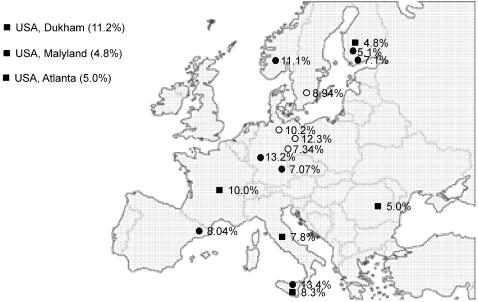
The prevalence of the nt13708 G/A polymorphism in Caucasian populations. The data are collected form 19 Caucasian population, including 6 published (closed square), 8 in this study (closed cycle) and 5 unpublished (open cycle). The locations are indicated on the map for the European populations. For the three USA populations, the location were indicated as the text. The prevalence of the mutation are presented as the percentage of the minor allele.

Since the nt13708 G/A variation seems to play a role in the susceptibility to MS and perhaps also in other complex diseases, it is important to consider ethnicities. A summary of the prevalence of the SNP in the Caucasian populations illustrates the point. So far, the prevalence of the nt13708A variant is known for 19 Caucasian populations, including 8 published populations [5], [9], [14], [21]–[24], 7 additional ones from this study and 4 own characterized unpublished results. The prevalence varied dramatically among populations, from 4.2% to 13.4% (Figure 2). A good example is the three populations in the USA of European ancestry, the prevalence varied from 4.8% to 11.2%.

In conclusion, we identified the nt13708A variant as susceptibility allele for MS. These findings could contribute to defining the role of the mitochondrial genome in MS pathogenesis. The mechanism underlying the association will be investigated in the future.

## Methods

### Subjects

Six European MS cohorts were recruited for this study. Three of them are well matched, in terms of age and geographic origin. These are the Spanish cohort from Barcelona with 424 patients and 517 unrelated healthy controls, the Norwegian cohort from Bergen with 390 patients and 190 unrelated healthy controls as well as a German cohort ( Germany I) from the Würzburg area with 285 patients and 382 unrelated healthy controls. The other three cohorts are not ideally matched. Those , include the cohorts from Finland, Germany (Germany II) and Sardinia. Population demographics of these three cohorts are summarized in Table 3. In total, 5,209 individuals (2,582 controls and 2,639 MS cases) were recruited for this study. All MS patients were classified according to the Poser criteria[31]. This study was approved by the local Ethical Committee.

### mtDNA genotyping

Seven common potentially functional mtDNA SNPs were selected for the association study, including 4 non-synonymous SNPs within protein coding region, 1 SNP within rRNA gene and 2 SNP within the control region. Genomic DNA was isolated from peripheral blood cells, using standard methods. All the samples were genotyped using the PCR-RFLP method with the exception of samples from Finnish cohort. The PCR amplification primers and restriction endonucleases used for the SNPs genotyping are summarized in supplementary Table S1. Amplification conditions are as follows: 95°C for 5 min, followed by 2 cycles of 94°C for 30 s, 57°C for 1 min, 72°C for 1 min, then another 36 cycles of 94°C for 30 s, 55°C for 1 min, 72°C for 1 min, and a final extension at 72°C for 7 min. The reactions were performed using GeneAmp PCR System 9700 cycler. 10 µl PCR products were digested by 1 u restriction endonuclease respectively at 37°C overnight. mtDNA SNPs were genotyped according to the size of the PCR products after the digestion. The SNP assay used to genotype the Finnish cohort was designed using SpectroDESIGNER (Sequenom), and the PCR and extension reactions were done as specified by the manufacturer. Genotypes were automatically called with the SpectroCALLER software (Sequenom), and manually checked as described in Silander et al [32].

### mtDNA sequencing and phylogenic analysis

The mtDNA were sequenced using microarray-based sequencing approach. The GeneChip® Human Mitochondrial Resequencing Array 2.0 were obtained from Affymetrix (Affymetrix, Inc. U.S.), with a sequence capacity of 16,535 bp (nt13 to nt16557). Genomic DNA with a concentration of 20ng/ul were used for the sequencing. PCR amplification, DNA fragmentation, labeling and chip hybridization were performed according Affymetrix CustomSeq Resequencing protocol. Fluorescent signals were collected by laser scan (GeneChip® Scanner 3000), and the data were analysed using GeneChip® Operating Software (GOS) and GeneChip® Sequence Analysis Software (GSEQ). The average call rate was 95.87 %. The mtDNA phylogenetic anlysis was performed using ClustalW software. To prevent the negative effect of the sites without call, we constructed the phylogenetic tree with only the polymorphic sites.

### Statistical analysis

We performed the association analysis for the case-cohort study using Comprehensive Meta-analysis Version 2 software (http://www.meta-analysis.com). For the individual MS cohorts, we calculated OR values with 95% CI and two-tailed P values using 2-by-2 contingency tables. We performed Mantel-Haenszel meta-analysis using a fixed model to calculate the OR and *P* values for the combined cohorts [33]. A *P* value of <0.05 was considered significant.

## Supporting Information

Table S1 (0.02 MB XLS)
